# Insulin Resistance Is Not Increased in Inflammatory Bowel Disease Patients but Is Related to Non-Alcoholic Fatty Liver Disease

**DOI:** 10.3390/jcm10143062

**Published:** 2021-07-10

**Authors:** Marta Carrillo-Palau, Alejandro Hernández-Camba, Noemi Hernández Alvarez-Buylla, Laura Ramos, Inmaculada Alonso-Abreu, Anjara Hernández-Pérez, Milagros Vela, Laura Arranz, Manuel Hernández-Guerra, Miguel Á. González-Gay, Iván Ferraz-Amaro

**Affiliations:** 1 Division of Gastroenterology, Hospital Universitario de Canarias, 38320 Tenerife, Spain; martacarry@yahoo.es (M.C.-P.); noehdz78@hotmail.com (N.H.A.-B.); laura7ramos@gmail.com (L.R.); macuaa@hotmail.com (I.A.-A.); anjarahdez@gmail.com (A.H.-P.); mhernand@ull.edu.es (M.H.-G.); 2 Division of Gastroenterology, Hospital Universitario de Nuestra Señora de la Candelaria, 38010 Tenerife, Spain; dr.alejandrohc@gmail.com (A.H.-C.); milvelillas@yahoo.es (M.V.); lauraarranz@gmail.com (L.A.); 3 Epidemiology, Genetics and Atherosclerosis Research Group on Systemic Inflammatory Diseases, Hospital Universitario Marqués de Valdecilla, IDIVAL, 39008 Santander, Spain; miguelggay@hotmail.com; 4 Division of Rheumatology, Hospital Universitario Marqués de Valdecilla, Universidad de Cantabria, 39008 Santander, Spain; 5 Cardiovascular Pathophysiology and Genomics Research Unit, School of Physiology, Faculty of Health Sciences, University of the Witwatersrand, Johannesburg 2000, South Africa; 6 Division of Rheumatology, Hospital Universitario de Canarias, 38320 Tenerife, Spain

**Keywords:** inflammatory bowel disease, insulin resistance, nonalcoholic fatty liver disease

## Abstract

Background. Insulin resistance (IR) has been linked to inflammatory states. The aim of this study was to determine whether IR is increased in a cohort of inflammatory bowel disease (IBD) patients with low disease activity. We additionally intended to establish which factors were the determinants of IR in this population, including the presence of nonalcoholic fatty liver disease (NAFLD). Methods. Cross-sectional study encompassing 151 IBD patients and 174 non-diabetic controls. Insulin and C-peptide serum levels and IR and beta cell function (%B) indices based on homoeostatic model assessment (HOMA2) were assessed in patients and controls. Liver stiffness as measured by transient elastography, and the presence of NAFLD detected via ultrasound were additionally assessed. A multivariable regression analysis was performed to evaluate the differences in IR indexes between patients and controls, and to determine which predictor factors were associated with IR in IBD patients. Results. Neither HOMA2-IR (beta coef. −0.26 {95%CI −0.64–0.13}, *p* = 0.19) nor HOMA2-%B (beta coef. 15 {95%CI −14–44}, *p* = 0.31) indexes differed between patients and controls after fully multivariable analysis. Among classic IR risk factors, obesity, abdominal circumference, and triglycerides significantly and positively correlated with IR indexes in IBD patients. However, most features related to IBD, such as disease patterns, disease activity, and inflammatory markers, were not associated with IR. The presence of NAFLD was independently and significantly associated with beta cell dysfunction in patients with IBD (HOMA2—B grade 4, 251 ± 40 vs. grade 1, 107 ± 37, *p* = <0.001). Conclusions. IR is not increased in IBD patients with low disease activity compared to controls. However, the presence of NAFLD favors the development of IR in patients with IBD.

## 1. Introduction

Insulin resistance (IR) refers to a state in which a given concentration of insulin is associated with a subnormal glucose response. It most commonly occurs in association with obesity, but may result from multiple underlying causes. In recent years, IR has been linked to inflammation [1]. In this sense, known systemic inflammatory diseases such as rheumatoid arthritis [2], spondyloarthritis [3], and systemic lupus erythematosus [4] have been associated with a higher degree of IR. Important long-term consequences of IR include the development of type 2 diabetes and cardiovascular disease.

Inflammatory bowel disease (IBD) is a chronic inflammatory state of the gastrointestinal tract that can be classified into two main categories: Crohn’s disease (CD) and ulcerative colitis (UC). Some controversy exists as to whether IBD is associated with IR. In this context, previous data have shown that IBD patients in a remission phase of the disease had whole-body glucose uptakes (as determined by the euglycemic hyperinsulinemic clamp) and IR indexes similar to those of control subjects [5,6]. In contrast, evidence from other studies suggests that inflammation contributes to IR in these patients [7].

Liver abnormalities are common in IBD and may occur due to the effects of the chronic inflammation present with the disease, or to the medications administered to these patients [8]. In this sense, there is a higher prevalence of nonalcoholic fatty liver disease (NAFLD) among IBD patients compared to the general population [9]. Similarly, liver stiffness—as measured by transient elastography—has been shown to be present in patients with IBD [10]. Liver fat and fibrosis have been associated with IR in the general population [11].

In the present study, we aimed to determine whether IR was higher in IBD patients compared to controls based on full multivariable analysis. We additionally intended to establish which factors are the determinants of IR in this population. Because hepatic disease has been found to be present in IBD, we further sought to establish whether liver abnormalities are related to IR in patients with IBD.

## 2. Methods

### 2.1. Study Participants

This was a cross-sectional study that included 151 consecutive patients with IBD and 174 controls. All participants were 18 years old or older, and IBD patients had a clinical diagnosis based upon clinical, endoscopic, and histological criteria during the previous 12 months. IBD patients had been diagnosed by a gastroenterologist and were periodically followed-up at gastroenterology outpatient clinics. For the purpose of inclusion in the present study, IBD disease duration had to be ≥1 year. Although long-term anti-tumor necrosis factor α therapy has been associated with improved IR [6,12], those undergoing anti-tumor necrosis factor α or other biological therapies were not excluded from the present study. The controls were community-based, and recruited by general practitioners in primary health centers. None of the patients or controls were under glucocorticoid treatment. Diabetes mellitus patients and controls were also excluded. Therefore, all patients and controls had glycemia < 110 mg/dL, and none were on glucose-lowering drugs or insulin therapy. Patients with alcoholic liver disease or hepatitis virus C infection were not included in this study. The study protocol was approved by the Institutional Review Committee at Hospital Universitario de Canarias and Hospital Universitario Nuestra Señora de La Candelaria, both in Spain, and all subjects provided informed written consent (approval no. CHUC_2019_103). The study protocol conformed to the ethical guidelines of the 1975 Declaration of Helsinki (6th revision, 2008) as reflected in a priori approval by the institution’s human research committee.

### 2.2. Data Collection

Surveys in IBD patients and controls were performed to assess cardiovascular risk factors and medication. Hypertension was defined as a systolic or diastolic blood pressure higher than 140 and 90 mmHg, respectively. Dyslipidemia was defined if one of the following factors was present: total cholesterol > 200 mg/dL, triglycerides > 150 mg/dL, high-density lipoprotein cholesterol (HDL) < 40 mg/dL in men or <50 mg/dL in women, or low-density lipoprotein cholesterol (LDL) > 130 mg/dL. Disease activity in CD was assessed using the Crohn’s Disease Activity Index (CDAI) and the Harvey-Bradshaw Index (HBI) [13]. CDAI was broken down into asymptomatic remission (0 to 149 points), mildly to moderately active (150 to 220 points), moderately to severely active (221 to 450 points), and severely active to fulminant disease (451 to 1100 points) categories as previously described [14]. Similarly, the Harvey-Bradshaw Index categorized remission as 0 to 4 points, mildly active disease as 5 to 7 points, moderately active disease as 8 to 16 points, and severely active disease as 17 to 100 points [13]. Disease activity in UC was calculated through the partial Mayo Clinic score [15].

### 2.3. Assessments

The homeostatic model assessment (HOMA) method was performed to determine IR. Briefly, the HOMA model enabled an estimation of insulin sensitivity (%S) and β-cell function (%B) based on fasting plasma insulin, C peptide, and glucose concentrations. In this study, we used HOMA2, the updated-computer HOMA model [16]. This model can be used to assess insulin sensitivity and beta cell function from paired fasting plasma glucose and specific insulin, or C peptide, concentrations over a range of 1–2200 pmol/L for insulin and 1–25 mmol/L for glucose. C peptide better estimates β-cell function as it is a marker of secretion; moreover, insulin data are preferable when calculating %S because HOMA-%S is derived from glucose disposal as a function of insulin concentration. In our study, IR and %S were calculated using insulin serum levels. Otherwise, %B was calculated using C-peptide serum levels. The computer model provided a value for insulin sensitivity expressed as HOMA2-%S (in which 100% is normal). HOMA2-IR (insulin resistance index) is simply the reciprocal of %S. Additionally, standard techniques were used to measure plasma glucose, C-reactive protein, serum lipids, and fecal calprotectin.

Abdominal ultrasonography was performed in patients with IBD in B mode in order to define the degrees of steatosis based on the existing fat infiltration. Fat infiltration was classified into three degrees as previously described [17,18]: mild, when there was a discreet diffuse increase in hepatic echogenicity, clearly displaying the diaphragmatic line and intrahepatic vascular structures; moderate, when intermediately hepatic echogenicity was observed compared to that of the kidney, as well as mean attenuation of the diaphragmatic wall and intrahepatic vessels, and severe, when there was a significant difference in hepatic and renal echogenicity, an absence of diaphragm visualization, and attenuation of vessels without being able to visualize them at the hepatic posterior pole. Transition elastography (ET) or Fibroscan^®^ was used to noninvasively establish the degree of hepatic fibrosis. Ten valid measurements were made, with a success rate greater than or equal to 60% and an interquartile range less than 30%, when deciding whether or not the result was valid. The degree of hepatic fibrosis was established according to F0 (no fibrosis) to F4 (cirrhosis) stages. Fibroscan^®^ values correlated with liver fibrosis as follows: <7.6 KPa = F0–F1, 7.7–9.4 KPa = F2, 9.5–14 KpPa = F3, >14 KPa = F4 [19,20]. Both abdominal ultrasound and elastography procedures were performed after 6 h of fasting.

### 2.4. Statistical Analysis

Demographic and clinical characteristics are shown as frequencies for binary variables. Continuous variables data are expressed as mean ± standard deviation (SD) or as median and interquartile ranges (IQR) for non-normally distributed variables. Univariable differences between patients and controls were assessed using T Student, U Mann–Whitney, Chi squared or Fisher Exact tests according to normal distributions or the number of subjects. Differences between patients and controls regarding IR indexes were assessed using multivariable linear regression analysis, adjusting for those variables with a *p* value less than 0.20 in their univariable differences. Any associations between disease-related features and liver abnormalities and IR indexes were assessed through linear multivariable regression analysis. All of the analyses used a 5% two-sided significance level and were performed using SPSS software, v. 25 (IBM, Chicago, IL, USA) and STATA software, v.15/SE (Stata Corp., College Station, TX, USA).

## 3. Results

### 3.1. Demographic, Laboratory, and Disease-Related Data

A total of 151 IBD patients and 174 controls with a mean ± SD age of 50 ± 16 and 48 ± 10 years, respectively, were included in this study. Demographic and disease-related characteristics of the participants are shown in Table 1. Body mass index (30 ± 3 vs. 27 ± 5 kg/m^2^, *p* = < 0.001) and waist circumference (98 ± 6 vs. 93 ± 24 cm, *p* = < 0.001) were higher in controls than in IBD patients. Regarding traditional cardiovascular risk factors, although the presence of hypertension, dyslipidemia, and current smoking did not differ between patients and controls, the latter were more frequently obese compare to those patients with IBD (40% vs. 28%, *p* = 0.034). Moreover, lipid profiles were similar between populations with the exception of LDL-cholesterol serum levels, which were significantly lower in IBD patients.

The median disease duration of IBD was 13 years (IQR 8–20). Patients with IBD were diagnosed with CD and UC types in 70% and 30% of cases, respectively. CD patients generally presented colonic and non-stricturing, non-penetrating types of disease. The median CDAI score was 38 (IQR 5–78), and 93% of the patients were classified as being in the asymptomatic-remission category. Similarly, the Harvey-Bradshaw index was 1 (IQR 0–3), and most of the patients (81%) were in the remission category of this index. Regarding UC, 49% were pancolitis, while 76% had a partial Mayo score of less than 2 points. Additional information regarding disease-related data is shown in Table 1.

### 3.2. Differences in Glucose Homeostasis Molecules and IR Indexes between Controls and Patients

Differences between glucose homeostasis molecules and IR indexes were adjusted for age, gender, body mass index, abdominal circumference, cholesterol serum levels, and the presence of hypertension and dyslipidemia. After this adjustment, IR indexes and C-peptide and insulin serum levels did not differ between patients with IBD and controls (Table 2).

### 3.3. Association of Disease-Related Data with Insulin Resistance and Beta Cell Function Indexes

The relationships between disease-related data and IR and beta cell function indexes are shown in Table 3. Traditional IR-related factors such as body mass index, abdominal circumference, and the presence of obesity were significantly and positively associated with both HOMA2-IR and -%B indexes. Moreover, the presence of hypertension was also associated with both indexes. CRP showed no relation to IR indexes. Regarding the lipid profile, some associations were found. In this sense, while triglycerides serum levels and the atherogenic index were significantly and positively related to both IR and beta cell function indexes, HDL-cholesterol was negatively associated with them.

Disease-specific patterns and features were, in general, not associated with IR or beta cell dysfunction. For example, disease duration, CD or UC patterns, CD disease activity scores (CDAI and Harvey-Bradshaw indexes), and treatments showed no relation to IR indexes. Only the partial Mayo score (beta coef. −24 {95%CI −45–−3}, *p* = 0.027) and the presence of perianal disease (beta coef. 39 {95%CI 12–65}, *p* = 0.005) were significantly related to HOMA2-%B after conducting a multivariable analysis (Table 3).

### 3.4. Liver Abnormalities in Relation to IR in Patients with IBD

Liver stiffness averaged 5.0 ± 2.0 kPa in IBD patients. Transient elastography was positively and significantly associated with insulin and C-peptide serum levels, and with IR and beta-cell function, in the univariable analysis. However, when these relations were adjusted for traditional factors related to both IR and liver disease, these associations were lost (Table 4). Besides, patients taking biological treatments did not show degrees of NAFLD different from those who did not.

Mild and moderate NAFLD grades were present, respectively, in 26% and 13% of the patients with IBD. As NAFLD grades increased, significantly higher insulin and C-peptide and IR indices were evident in the univariable analysis. When this analysis was adjusted for confounders, some associations remained significant. In this sense, patients with severe fat infiltration (grade 4) had higher serum levels of insulin and C-peptide, as well as higher HOMA2-IR and HOMA2-%B indices. Moreover, adjusted-trend analysis showed significant values for C-peptide and HOMA2-%B, indicating that as NAFLD grades increased, values for both C-peptide and HOMA2-%B rose as well (Table 4). Moreover, the addition of IBD type as an interaction factor in the regression analysis yielded no significant values, showing that this relation did not differ between the two IBD types (Table 4).

## 4. Discussion

IR has been linked to inflammatory states. In this regard, increased IR has been described in patients with inflammatory diseases such as rheumatoid arthritis and systemic lupus erythematosus [2,4]. However, our data do not support that it may be the same for IBD, in particular in patients with low disease activity, despite the fact that it is also considered a disease with a high inflammatory component. In this regard, in our study, CRP was not higher in patients compared to controls. Furthermore, most of the patients presented low disease activity scores. For this reason, we cannot rule out the possibility that patients with a higher burden of disease activity might present a higher IR. However, our findings indicate that the presence of the disease itself, regardless of its activity, is not associated with increased IR.

Previous studies about IR in IBD are scarce, and most have lacked statistical power because the numbers of patients recruited have been, in general, low. For example, a previous study involving 40 patients with IBD and 40 controls reported a higher IR in patients [21]. However, this work did not include a multivariable adjustment, C-peptide was not assessed, and the IR calculation was not made using the updated HOMA2 model, which takes account of variations in hepatic and peripheral glucose resistance. Similarly, a study involving 17 patients with CD showed that they had significantly higher IR indexes than did the controls [22]. Interestingly, patients with active disease (CDAI ≥ 150) showed significantly lower values for HOMA (i.e., had a lower degree of IR) than those with inactive disease (CDAI < 150). Regarding this finding, the authors argued against chronic inflammation as a possible cause for IR. The low recruitment of patients in this study did not allow for a multivariable analysis. Similarly, in a previous study involving 102 IBD patients without cardiovascular risk factors and 74 matched controls, IR calculated via the non-updated HOMA index was found to be higher in IBD [7]. However, again, this report lacked a multivariable adjustment. In another study of 20 patients with IBD and 40 controls, no differences in IR were found [5]. Our work is the first to recruit a large number of patients for the study of IR in IBD. Moreover, our study assessed full glucose homeostasis molecules, including C-peptide. Furthermore, our sample size allowed for a multivariable analysis and control of confounding factors. For this reason, we believe that the reported conclusions in the current work are of greater strength than those of previous studies.

In our study, disease-related factors such as disease duration, disease phenotypes, CD activity, and/or the use of different treatments were not associated with IR indexes. Only some significant associations were found after the multivariable analysis. In this sense, while CU’s Mayo score was negatively associated with beta cell function, the presence of perianal disease was positively related to it. In contrast, traditional IR factors such as obesity, abdominal circumference, body mass index, and lipid profile were significantly and highly associated with both IR and beta cell function indexes. For this reason, we believe that IR in these patients was driven by these classic factors associated with IR and that the disease, through its different features, was not influencing the presence of IR.

Strong epidemiological, biochemical, and therapeutic evidence supports the premise that the primary pathophysiological derangement in most patients with NAFLD is IR [23]. Insulin resistance leads to increased lipolysis, triglyceride synthesis, increased hepatic uptake of free fatty acids, and accumulation of hepatic triglyceride. It is known that IR is a characteristic feature of NAFLD even when subjects are not obese or have normal glucose tolerance. On the other hand, NAFLD is highly prevalent among patients with type 2 diabetes (up to 70%) that show increased hepatic triglyceride accumulation independently of BMI. Insulin-resistant subjects with NAFLD show reduced insulin sensitivity not only at the level of the muscle but also at the level of the liver and adipose tissue [24].

On the other hand, the liver plays a central role in the systemic regulation of glucose, and aberrant hepatic insulin action is thought to be a primary driver of IR [25]. Therefore, it is thought that liver disease is an important source of IR in the general population. IBD is accompanied by liver injury, which can include the presence of NAFLD and fibrosis. In this context, sonographic evidence of fatty liver tissue is not an uncommon finding in patients with IBD, In fact, a recent study of 511 IBD patients showed a prevalence of up to 35% [26], while liver fibrosis (as detected by transient elastography) was also found to be present [10]. In keeping with the aforementioned concepts, we sought to determine whether IR could be related to those liver abnormalities that might potentially be present in IBD. In our study, NAFLD was associated with beta cell dysfunction after the multivariable analysis. We therefore believe that NAFLD is adding IR to the disease, but not to the point of causing higher levels than in controls.

We acknowledge the limitation that we did not assess NAFLD or liver fibrosis in controls. Nevertheless, we were interested in how both of these were related to IR in IBD patients compared to controls. If we had had such data for controls, we could have studied whether the effect of liver disease on IR differed between patients and controls.

In conclusion, unlike patients with other inflammatory states, IBD patients with low disease activity do not present increased IR compared to the general population. NAFLD and classic factors associated with metabolic syndrome account for the presence of IR in these patients.

## Figures and Tables

**Table 1:** Characteristics of IBD patients and controls.

		Controls	IBD Patients	
		(*n* = 174)	(*n* = 151)	*p*
	Age, years	50 ± 16	48 ± 10	0.10
	Male, *n* (%)	56 (32)	65 (43)	**0.043**
	Body mass index, kg/m^2^	30 ± 3	27 ± 5	**<0.001**
	Abdominal circumference, cm	98 ± 6	93 ± 12	**<0.001**
	Systolic blood pressure, mmHg	129 ± 10	125 ± 18	**0.011**
	Diastolic blood pressure, mmHg	75 ± 8	74 ± 11	0.13
Cardiovascular co-morbidity				
	Smoking, *n* (%)	34 (20)	25 (17)	0.49
	Hypertension, *n* (%)	49 (28)	26 (17)	0.20
	Dyslipidemia, *n* (%)	113 (65)	109 (72)	0.16
	Type 2 diabetes, *n* (%)	0 (0)	0 (0)	-
	Obesity, *n* (%)	69 (40)	43 (28)	**0.034**
Analytical and lipid profile				
	CRP, mg/L	1.8 (1.0–3.9)	1.71 (0.9–3.9)	0.56
	Cholesterol, mg/dL	204 ± 42	196 ± 44	0.11
	Triglycerides, mg/dL	138 ± 73	141 ± 80	0.74
	HDL cholesterol, mg/dL	54 ± 15	55 ± 16	0.73
	LDL cholesterol, mg/dL	122 ± 34	113 ± 37	**0.026**
	Atherogenic index	3.97 ± 1.10	3.81 ± 1.19	0.21
IBD-related data				
Crohn’s disease, *n* (%)			105 (70)	
Ulcerative colitis, *n* (%)			46 (30)	
Disease duration since diagnosis, years			13 (8–20)	
Crohn’s disease-related data, *n* (%)				
	A1 below 16 years		16 (15)	
	A2 between 17 and 40 years		65 (62)	
	A3 above 40 years		23 (22)	
	L1 ileal		40 (38)	
	L2 colonic		21 (20)	
	L3 ileocolonic		43 (41)	
	L4 isolated upper disease		10 (10)	
	B1 non-stricturing, non-penetrating		58 (55)	
	B2 stricturing		35 (33)	
	B3 penetrating		12 (11)	
	CDAI score		38 (5–78)	
	Asymptomatic remission		93 (89)	
	Mildly to moderately active CD		8 (8)	
	Moderately to severely active CD		3 (3)	
	Severely active to fulminant disease		0 (0)	
	Harvey-Bradshaw Index		1 (0–3)	
	Clinical remission		90 (86)	
	Mildly active disease		9 (9)	
	Moderately active disease		4 (4)	
	Severely active disease		1 (1)	
Ulcerative colitis-related data, *n* (%)				
	Proctosigmoiditis		6 (13)	
	Left-sided colitis		17 (37)	
	Pancolitis		21 (46)	
	Partial Mayo score		1 (0–1)	
	<2		38 (83)	
	≥2		8 (17)	
Fecal calprotectin, mcg/g				
	<300		104 (69)	
	≥300		19 (13)	
Perianal disease, *n* (%)			14 (9)	
Previous surgery, *n* (%)			44 (29)	
Extraintestinal manifestations				
	Arthritis, *n* (%)		4 (3)	
	Uveitis, *n* (%)		3 (2)	
	Erythema nodosum, *n* (%)		2 (1)	
	Psoriasis, *n* (%)		2 (1)	
Oral mesalazine, *n* (%)			53 (35)	
Methotrexate, *n* (%)			20 (13)	
Azathioprine, *n* (%)			47 (31)	
Anti-TNF therapy, *n* (%)			48 (32)	
	Adalimumab, *n* (%)		20 (13)	
	Infliximab, *n* (%)		28 (19)	
Ustekinumab, *n* (%)			6 (4)	
Vedolizumab, *n* (%)			5 (3)	

Data represent mean ± SD or median (interquartile range) when data were not normally distributed. BMI: body mass index; CRP: C-reactive protein; LDL: low-density lipoprotein. HDL: high-density lipoprotein; TNF: tumor necrosis factor; CDAI: Crohn’s Disease Activity Index. Dyslipidemia was defined if one of the following was present: total cholesterol > 200 mg/dL, triglyceride > 150 mg/dL, HDL cholesterol < 40 in men or <50 mg/dL in women, or LDL cholesterol > 130 mg/dL. CDAI was categorized as 0 to 149: Asymptomatic remission; 150 to 220 points: Mildly to moderately active; 221 to 450 points: Moderately to severely active; 451 to 1100 points: Severely active to fulminant disease. Harvey-Bradshaw Index was categorized as 0 to 4 points: Clinical remission; 5 to 7 points: Mildly active disease 8 to 16 points: Moderately active disease; 17 to 100 points: Severely active disease. Disease-related data percentages refer to the number of each of the IBD types. Significant *p*-values are depicted in bold.

**Table 2:** Multivariable regression analysis of the differences between controls and patients in terms of glucose homeostasis molecules and IR indexes.

	Beta Coef. (95% CI), *p*
Insulin, µU/mL	−1.74 (−4.76–1.28), 0.26
C-peptide, ng/mL	−0.39 (−1.10–0.31), 0.27
HOMA2-IR	−0.26 (−0.64–0.13), 0.19
HOMA2-S%	17 (−31–66), 0.48
HOMA2-B%-C-peptide	15 (−14–44), 0.31

Beta coefficients are expressed using controls as the reference category. Controls and patients are considered the independent variables. *p*’ values are adjusted for age, gender, body mass index, abdominal circumference, cholesterol serum levels, and the presence of hypertension and dyslipidemia. HOMA2-IR and -S%: Homeostatic assessment model for the assessment of insulin resistance using insulin and glucose serum levels. HOMA2%B-C peptide: Homeostatic assessment model for the assessment of beta cell function using C peptide and glucose serum levels.

**Table 3:** Disease-related data association with insulin resistance and beta cell function indexes in IBD patients.

		Beta Coef. (95%CI), *p*			
		HOMA2-IR	*p* *	HOMA2-%B	*p* *
	Age, years	0.00 (−0.01–0.01), 0.52		0.44 (−0.33–1.21), 0.26	
	Male	0.05 (−0.15–0.26), 0.61		0.49 (−15.43–16.41), 0.95	
	Body mass index, kg/m^2^	**0.06 (0.04–0.08), <0.001**		**4 (3–6), <0.001**	
	Abdominal circumference, cm	**0.02 (0.02–0.03), <0.001**		**2 (1–2), <0.001**	
	Systolic blood pressure, mmHg	0.01 (0.00–0.01), 0.10		0.39 (−0.05–0.82), 0.082	
	Diastolic blood pressure, mmHg	**0.01 (0.00–0.02), 0.044**		0.40 (−0.31–1.11), 0.27	
Cardiovascular co-morbidity					
	Smoking	0.05 (−0.23–0.32), 0.74		14 (−7–36), 0.18	
	Hypertension	**0.48 (0.22–0.74), <0.001**		**44 (24–63), <0.001**	
	Dyslipidemia	0.19 (−0.04–0.42), 0.097		0.74 (−16.85–18.34), 0.93	
	Obesity	**0.60 (0.39–0.81), <0.001**		**48 (33–64), <0.001**	
Laboratory and lipid profile					
	CRP, mg/L	0.01 (−0.01–0.03), 0.48		1 (0–3), 0.12	
	Cholesterol, ×10 mg/dL	0.00 (−0.03–0.02), 0.78		−2 (−3–0), 0.063	
	Triglycerides, ×10 mg/dL	**0.04 (0.03–0.05), <0.001**		**2 (2–3), <0.001**	
	HDL cholesterol, ×10 mg/dL	−0.01 (−0.18–−0.05), <0.001		−1 (−2–−1), <0.001	
	LDL cholesterol, ×10 mg/dL	−0.02 (−0.05–0.01), 0.19		**−2 (−5–−0), 0.020**	
	Atherogenic index	**0.21 (0.13–0.29), <0.001**		**14 (8–20), <0.001**	
IBD-related data					
Crohn’s disease		−0.17 (−0.39–0.08), 0.14	0.95	−11 (−28–6), 0.19	0.75
Ulcerative colitis					
Disease duration, years		0.00 (−0.01–0.01), 0.89		0.40 (−0.45–1.24), 0.35	
Crohn’s disease-related data					
	A1 below 16 years	0.06 (−0.30–0.42), 0.73		−2 (−29–26), 0.91	
	A2 between 17 and 40 years	0.08 (−0.18–0.34), 0.54		2 (−18–22), 0.84	
	A3 above 40 years	−0.15 (−0.46–0.16), 0.33		−2 (−26–22), 0.87	
	L1 ileal	−0.09 (−0.35–0.18), 0.51		−11 (−32–9), 0.27	
	L2 colonic	−0.13 (−0.45–0.19), 0.43		−11 (−36–13), 0.36	
	L3 ileocolonic	0.21 (−0.04–0.47), 0.10	0.26	**22 (3–42), 0.027**	0.061
	L4 isolated upper disease	−0.26 (−0.69–0.18), 0.25		−8 (−42–26), 0.64	
	B1 non-stricturing, non-penetrating	−0.03 (−0.28–0.22), 0.81		2 (−17–22), 0.82	
	B2 stricturing	0.07 (−0.20–0.34), 0.62		7 (−14–27), 0.54	
	B3 penetrating	0.05 (−0.36–0.46), 0.82		−12 (−44–19), 0.43	
	*log* CDAI score	−0.13 (−0.37–0.11), 0.29		−4 (−26–17), 0.69	
	Asymptomatic remission	ref.		ref.	
	Mildly to moderately active	−0.18 (−0.67–0.31), 0.46		−12 (−49–26), 0.55	
	Moderately to severely active	−0.37 (−1.15–0.41), 0.35		−8 (−68–52), 0.79	
	Severely active to fulminant	-		-	
	*log* Harvey-Bradshaw Index	−0.08 (−0.24–0.09), 0.38		0.42 (−12.56–13.40), 0.95	
	Clinical remission	ref.		ref.	
	Mildly active disease	0.05 (−0.41–0.52), 0.82		12 (−24–47), 0.52	
	Moderately active disease	0.36 (−1.04–0.33), 0.30		4 (−48–56), 0.88	
	Severely active disease	−0.38 (−1.72–0.96), 0.58		−62 (−165–41), 0.23	
Ulcerative colitis-related data					
	Proctosigmoiditis	−0.38 (−0.85–0.09), 0.11	0.28	**−38 (−73–−2), 0.038**	0.26
	Left-sided colitis	−0.11 (−0.45–0.23), 0.53		13 (−13–38), 0.34	
	Pancolitis	0.24 (−0.08–0.56), 0.14	0.52	3 (−23–28), 0.84	
	*log* Partial Mayo score	−0.17 (−0.45–0.12), 0.24		**−24 (−45–−3), 0.027**	**0.048**
	<2	ref.		ref.	
	≥2	−0.27 (−0.69–0.15), 0.20	0.14	−22 (−55–11), 0.19	0.080
Fecal calprotectin, mcg/g					
	<300	ref.		ref.	
	≥300	−0.25 (−0.58–0.08), 0.13		1 (−24–26), 0.95	
Perianal disease		0.31 (−0.04–0.66), 0.080	0.42	**39 (12–65), 0.005**	**0.024**
Previous surgery		0.10 (−0.12–0.33), 0.37		7 (−11–24), 0.45	
Extraintestinal manifestations					
	Arthritis	−0.16 (−0.80–0.48), 0.63		−31 (−80–18), 0.21	
	Uveitis	0.08 (−0.66–0.82), 0.83		−3 (−60–53), 0.90	
	Erythema nodosum	−0.18 (−1.08–0.71), 0.69		38 (−30–107), 0.27	
	Psoriasis	−0.15 (−1.05–0.75), 0.75		−5 (−74–64), 0.89	
Oral mesalazine		0.11 (−0.11–0.32), 0.32		11 (−5–28), 0.18	0.11
Methotrexate		−0.04 (−0.34–0.27), 0.81		−2 (−25–21), 0.85	
Azathioprine		0.04 (−0.18–0.26), 0.73		−12 (−30–4), 0.14	0.28
Anti-TNF therapy		0.13 (−0.09–0.35), 0.26		9 (−8–26), 0.31	
	Adalimumab	−0.03 (−0.33–0.28), 0.87		8 (−15–32), 0.47	
	Infliximab	0.20 (−0.06–0.46), 0.14	0.64	6 (−14–26), 0.56	
Ustekinumab		−0.17 (−0.69–0.36), 0.53		−3 (−43–38), 0.88	
Vedolizumab		−0.14 (−0.72–0.43), 0.62		−9 (−53–35), 0.68	

Data represent mean ± SD or median (interquartile range) when data were not normally distributed. BMI: body mass index; CRP: C-reactive protein; LDL: low-density lipoprotein; CDAI: Crohn’s Disease Activity Index. HDL: high-density lipoprotein; TNF: tumor necrosis factor. Dyslipidemia was defined if one of the following was present: total cholesterol > 200 mg/dL, triglyceride > 150 mg/dL, HDL cholesterol < 40 in men or <50 mg/dL in women, or LDL cholesterol > 130 mg/dL. CDAI was categorized as 0 to 149: Asymptomatic remission; 150 to 220 points: Mildly to moderately active; 221 to 450 points: Moderately to severely active; 451 to 1100 points: Severely active to fulminant disease. Harvey-Bradshaw Index was categorized as 0 to 4 points: Clinical remission; 5 to 7 points: Mildly active disease; 8 to 16 points: Moderately active disease; 17 to 100 points: Severely active disease. Beta coef. inferior to 1 are expressed in decimals, otherwise in integer numbers. HOMA2IR: Homeostatic assessment model for the assessment of insulin resistance using insulin and glucose serum levels. HOMA2%B-C peptide: Homeostatic assessment model for the assessment of beta cell function using C peptide and glucose serum levels. Univariable relations with a *p* value inferior to 0.20 were further analyzed and adjusted for body mass index, abdominal circumference, hypertension, obesity, triglycerides, and HDL cholesterol. Ref. denotes reference category (* represents *p* values adjusted for covariables). Significant *p*-values are depicted in bold.

**Table 4:** Relation of fibroscan and NAFLD to glucose homeostasis-related molecules and IR indexes.

			Glucose, mg/dL			Insulin, U/mL			C-peptide, ng/mL			HOMA2-IR			HOMA2-B%		
			Beta Coef. (95% CI), *p*														
Fibroscan, kPa																	
	5.0 ± 2.0		−0.36 (−1.18–0.46)	0.38		**0.46 (0.09–0.84)**	**0.017**		**0.11 (0.03–1.91)**	**0.010**		**0.06 (0.01–0.11)**	**0.023**		**6 (3–10)**	**0.001**	
	Adjusted *		−0.36 (−1.20–0.48)	0.40		0.08 (−0.27–0.43)	0.64		0.03 (−0.05–0.10)	0.51		0.01 (−0.04–0.05)	0.72		3 (−1–6)	0.11	
Ultrasound grade of steatosis																	
			Mean ± standard deviation, *p*														
	Grade	*n*		*p*	*p* **		*p*	*p* **		*p*	*p* **		*p*	*p* **		*p*	*p* **
	0	73	89 ± 9	-	-	6 ± 4	-	-	1.6 ± 0.9	-	-	0.8 ± 0.5	-	-	107 ± 37	-	-
	1	40	89 ± 10	0.92	0.95	9 ± 5	**<0.001**	0.18	2.3 ± 1.0	**<0.001**	0.28	1.2 ± 0.6	**<0.001**	0.18	137 ± 42	**<0.001**	0.17
	2	19	88 ± 10	0.49	0.073	10 ± 5	**0.009**	0.77	2.6 ± 1.3	**0.001**	0.52	1.3 ± 0.6	**<0.001**	0.73	153 ± 57	**<0.001**	**0.040**
	3	4	85 ± 6	0.37	0.29	19 ± 13	**<0.001**	**0.006**	4.7 ± 1.5	**0.002**	**0.000**	2.4 ± 1.6	**0.010**	**0.011**	251 ± 40	**<0.001**	**<0.001**
Trend *p*				0.34	0.10		**<0.001**	0.18		**<0.001**	**0.024**		**<0.001**	0.22		**<0.001**	**0.001**
CD x CU Interaction *p*					0.46			0.84			0.59			0.75			0.96

Fibroscan and ultrasound grades are considered the independent variables. Ultrasound steatosis grades were 0: absent, 1: mild; 2: moderate; 3: severe. HOMA2IR: Homeostatic assessment model for the assessment of insulin resistance using insulin and glucose serum levels. HOMA2%B-C peptide: Homeostatic assessment model for the assessment of beta cell function using C peptide and glucose serum levels. CD: Crohn’s disease; UC: Ulcerative colitis. Significant *p* values are depicted in bold. * Adjusted for abdominal circumference, HDL cholesterol and triglycerides. ** Adjusted for BMI, abdominal circumference, hypertension obesity, triglycerides, and HDL-cholesterol.

## Data Availability

The data presented in this study are available on request to the corresponding author.
